# Characterization of *MED12*, *HMGA2*, and *FH* alterations reveals molecular variability in uterine smooth muscle tumors

**DOI:** 10.1186/s12943-017-0672-1

**Published:** 2017-06-07

**Authors:** Netta Mäkinen, Kati Kämpjärvi, Norma Frizzell, Ralf Bützow, Pia Vahteristo

**Affiliations:** 1 Research Programs Unit, Genome-Scale Biology Research Program and Medicum, Department of Medical and Clinical Genetics, FIN-00014 University of Helsinki, P.O. Box 63, Helsinki, Finland; 2 Department of Pharmacology, Physiology and Neuroscience, USC School of Medicine, University of South Carolina, Columbia, SC 29209 USA; 3 Department of Pathology, Laboratory of Helsinki University Hospital (HUSLAB), Helsinki University Hospital and Medicum, FIN-00014 University of Helsinki, P.O. Box 21, Helsinki, Finland

**Keywords:** Uterine leiomyoma, Histopathological uterine leiomyoma variants, Uterine leiomyosarcoma, *MED12*, *HMGA2*, *FH*

## Abstract

Uterine smooth muscle tumors range from benign leiomyomas to malignant leiomyosarcomas. Based on numerous molecular studies, leiomyomas and leiomyosarcomas mostly lack shared mutations and the majority of tumors are believed to develop through distinct mechanisms. To further characterize the molecular variability among uterine smooth muscle tumors, and simultaneously insinuate their potential malignant progression, we examined the frequency of known genetic leiomyoma driver alterations (*MED12* mutations, *HMGA2* overexpression, biallelic *FH* inactivation) in 65 conventional leiomyomas, 94 histopathological leiomyoma variants (18 leiomyomas with bizarre nuclei, 22 cellular, 29 highly cellular, and 25 mitotically active leiomyomas), and 51 leiomyosarcomas. Of the 210 tumors analyzed, 107 had mutations in one of the three driver genes. No tumor had more than one mutation confirming that all alterations are mutually exclusive. *MED12* mutations were the most common alterations in conventional and mitotically active leiomyomas and leiomyosarcomas, while leiomyomas with bizarre nuclei were most often FH deficient and cellular tumors showed frequent *HMGA2* overexpression. Highly cellular leiomyomas displayed the least amount of alterations leaving the majority of tumors with no known driver aberration. Our results indicate that based on the molecular background, histopathological leiomyoma subtypes do not only differ from conventional leiomyomas, but also from each other. The presence of leiomyoma driver alterations in nearly one third of leiomyosarcomas suggests that some tumors arise through leiomyoma precursor lesion or that these mutations provide growth advantage also to highly aggressive cancers. It is clinically relevant to understand the molecular background of various smooth muscle tumor subtypes, as it may lead to improved diagnosis and personalized treatments in the future.

**Electronic supplementary material:**

The online version of this article (doi:10.1186/s12943-017-0672-1) contains supplementary material, which is available to authorized users.

## Background

Uterine leiomyomas (ULs) are extremely common human neoplasms that originate from the smooth muscle cells of the myometrium. Due to their dependency on the ovarian steroids estrogen and progesterone, ULs occur in women of reproductive age and typically regress with the onset of menopause [1]. The life time prevalence of ULs is approximately 70% among white and more than 80% among black women [2]. Despite their benign nature, ULs can cause considerable morbidity, such as heavy and prolonged menstrual flow, abdominal pain and discomfort, and reproductive dysfunction. They are also the single most prevalent cause for hysterectomy and pose a considerable socio-economic impact [3]. Based on histopathology, ULs can be divided into conventional leiomyomas and various relatively rare subtypes, such as leiomyomas with bizarre nuclei, cellular, and mitotically active leiomyomas [4]. Histopathological UL subtypes explain approximately 10% of all ULs. Although these subtypes mimic malignancy in one or more aspects, their behavior is thought to be benign.

Approximately 40% of leiomyomas harbor non-random cytogenetic rearrangements, of which the most common is a translocation between chromosome bands 12q15 and 14q24 leading to overexpression of *high mobility group AT-hook 2* (*HMGA2*) [5–7]. Other chromosomal alterations include, for example, interstitial deletions in 7q, rearrangements of 6p21, and trisomy 12. Several independent studies representing various ethnic groups have shown that approximately 70% of ULs harbor specific mutations in *mediator complex subunit 12* (*MED12)* [8–11]. All observed changes have been missense or small in-frame insertion-deletion mutations in exons 1 and 2, affecting a highly conserved region of the gene. Subsequent studies have indicated that histopathological UL subtypes harbor significantly fewer *MED12* mutations than conventional leiomyomas [12, 13]. Rarely, ULs can be associated with a familial Hereditary Leiomyomatosis and Renal Cell Cancer (HLRCC) syndrome. The syndrome is caused by heterozygous germline mutations in *fumarate hydratase* (*FH*), which encodes an enzyme fumarase of tricarboxylic acid cycle [14]. Our recent high-throughput sequencing efforts have pointed to at least three distinct molecular subclasses among conventional ULs, each candidate subclass displaying a characteristic genetic driver aberration as well as unique global gene expression profile: *MED12* mutation-positive, *HMGA2*-overexpressing, and FH-deficient ULs [15, 16].

Uterine leiomyosarcomas (ULMSs) are rare, malignant smooth muscle tumors with a poor 5-year survival and high recurrence rate [17–20]. The majority of ULMSs occur in women >50 years of age with an annual incidence of 0.3–0.4/100,000 women [21, 22]. The presenting symptoms of this tumor type greatly overlap with those of benign ULs, making early diagnosis of ULMSs difficult. Surgery is the recommended primary treatment, yet the diagnosis is often made histologically after the surgery. Even then, the clinical course of ULMS is difficult to predict. Occasionally, diagnostic challenges emerge in daily pathological practice in distinguishing ULMSs from histopathological UL subtypes. The accurate diagnosis has important prognostic and therapeutic implications. Most ULMSs are aneuploid with complex structural chromosomal alterations [23], however, no consistent structural aberrations have been identified in these tumors. So far, only a few genes have been associated with ULMSs, including *TP53*, *RB1*, *ATRX*, and *MED12* [23, 24].

The aim of this study was to examine the frequency of known genetic leiomyoma driver alterations in histopathological UL subtypes and ULMSs to scrutinize molecular variability in these tumors and to identify a potential UL subtype(s) that resembles ULMSs. The study material consisted of a comprehensive series of 94 histopathological UL variants (incl. leiomyomas with bizarre nuclei, cellular, and mitotically active leiomyomas), 51 ULMSs, and 65 conventional ULs as controls. *MED12* mutation status was analyzed by direct sequencing, while *HMGA2* overexpression and biallelic *FH* inactivation were determined by immunohistochemistry.

## Methods

### Samples

A retrospective series of 210 archival formalin-fixed paraffin-embedded (FFPE) samples representing various uterine smooth muscle tumors was collected at the Department of Pathology, Hospital District of Helsinki and Uusimaa, Helsinki, Finland, after which the samples were anonymized for the study. The series included 65 conventional, 51 cellular (22 cellular and 29 highly cellular), and 25 mitotically active leiomyomas (11 lesions showing simultaneously increased cellularity), 18 leiomyomas with bizarre nuclei, and 51 leiomyosarcomas.

### Histological evaluation

Histological assessment of each specimen was performed by a pathologist specialized in gynecological pathology (RB) and the tumors were classified into conventional, cellular, highly cellular, and mitotically active leiomyomas, leiomyomas with bizarre nuclei, and ULMSs according to the WHO criteria [4] (see Additional file 1: Table S1). The microscopic morphology of highly cellular leiomyoma may mimic endometrial stromal tumor. In ambiguous cases, the differential diagnosis was aided by smooth muscle actin, desmin, h-caldesmon (smooth muscle markers), and CD-10 (relative endometrial stromal cell marker) immunohistochemistry. For DNA extraction and tissue microarray (TMA) construction, pathologist marked representative tumor regions of each sample.

### DNA extraction and tissue microarray construction

Genomic DNA was extracted either with NucleoSpin® FFPE DNA Kit, NucleoSpin® FFPE RNA/DNA Kit (Macherey-Nagel, Düren, Germany), or with a conventional non-enzymatic method [25]. Quadruplicate 0.8 mm cores of FFPE tumor tissue from each case were punched with a manual tissue arrayer (MTA-I, Beecher Instruments, Sun Prairie, WI, USA) to an empty paraffin block. Cores of FFPE myometrium tissue were also incorporated in the TMA block as internal controls.

### *MED12* mutation analysis

*MED12* mutation screening of exons 1 and 2 was performed by direct sequencing. Exon 2, which harbors the great majority of mutations, was analyzed first and if negative, mutation screening was extended to exon 1. *MED12* exon 2 mutation status has been previously reported for 64 out of 65 conventional ULs, 91 out of 94 histopathological UL variants, and 19 out of 51 ULMSs [12, 24, 26], and exon 1 for all *MED12* exon 2 mutation-negative conventional ULs (*n* = 28) [26] and histopathological UL variants (*n* = 77) [27]. The primer sequences and conditions have been previously described [12, 27]. Sequencing was carried out using Big Dye Terminator v3.1 sequencing chemistry (Applied Biosystems, Foster City, CA, USA) on an ABI3730 automatic DNA Sequencer according to the manufacturer’s instructions. The sequence graphs were analyzed both manually and with Mutation Surveyor software (SoftGenetics, State College, PA, USA). *MED12* NM_005120.2 was used as a reference sequence. *MED12* mutation screening was successful in 98.6% (207/210) of the samples for exon 2 and in 95.2% (138/145) for exon 1. The three samples which failed in direct sequencing of exon 2 showed clearly visible larger deletions on an agarose gel (see Additional file 2: Figure S1).

### Immunohistochemistry

*HMGA2* overexpression and biallelic *FH* inactivation were assessed by immunohistochemistry. Biallelic inactivation of *FH* was detected with 2-succinocysteine (2SC) staining, which is based on the recognition of modified (succinated) proteins formed in FH-deficient cells as a result of the accumulation of fumarate [28, 29]. 2SC status has been previously reported for all 65 conventional ULs [26]. Heat-induced antigen retrieval was carried out in a microwave using citrate buffer (pH 6.0). Endogenous peroxidase blocking was followed by overnight incubation with the primary antibody at 4 °C (anti-HMGA2 1:2000, Biocheck Inc., Foster City, CA, USA; anti-2SC 1:2000 [29]). A positive reaction for HMGA2 and 2SC was detected by DAB Plus Substrate System (Thermo Fisher Scientific, Waltham, MA, USA) or EnVision + kit (Dako, Carpinteria, CA, USA), respectively.

### Scoring of the TMAs

Immunohistochemical scoring was carried out by a pathologist (RB). For both HMGA2 and 2SC, the intensity of the immunoreaction was classified into four groups: - = fully negative, (+) = single cell positivity, + = low positivity, ++ = strong positivity (see Additional file 3: Figure S2). Only samples that showed strong positivity in the immunoreaction were considered positive. For 2SC, the positive staining indicated accumulation of fumarate and succinated proteins, and thus biallelic inactivation of *FH*, while the negative staining indicated that the cells retained sufficient amount of FH. For HMGA2, only nuclear labelling of the protein was evaluated.

### *FH* mutation analysis

Tumors showing 2SC positivity (*n* = 10) were directly sequenced for *FH* mutations. The sequencing was carried out as described above in *MED12* mutation analysis. *FH* NM_000143.3 was used as a reference sequence. *FH* exon 1 was excluded from the mutation screening. Two out of ten 2SC-positive tumors, both representing ULMSs, did not amplify at all and were thus excluded from the mutation screening.

### Statistical analyses

Statistical analyses were performed using R software, version 2.14.0 (R Foundation for Statistical Computing, Vienna, Austria, www.r-project.org). Fisher’s exact test was used to compare the frequency of *MED12* mutations, presence of *HMGA2* overexpression and biallelic *FH* inactivation between conventional ULs and histopathological UL subtypes or ULMSs. Eleven mitotically active leiomyomas with increased cellularity were included in both mitotically active and cellular/highly cellular leiomyoma subtypes for statistical testing. All *P*-values were two-sided and *P*-value <0.05 was considered statistically significant.

## Findings

### Uterine smooth muscle tumor classification

Based on the number of mitotic figures per 10 high power fields, severity of nuclear atypia (0–3), degree of cellularity (normal, cellular, highly cellular), and presence of tumor necrosis in the hematoxylin-eosin-stained sections of the tumor specimens, the samples were classified into 65 conventional, 51 cellular (22 cellular and 29 highly cellular), and 25 mitotically active leiomyomas (11 lesions showing simultaneously increased cellularity), 18 leiomyomas with bizarre nuclei, and 51 leiomyosarcomas (see Additional file 4: Figure S3).

### Conventional leiomyomas

**Fig. 1:**
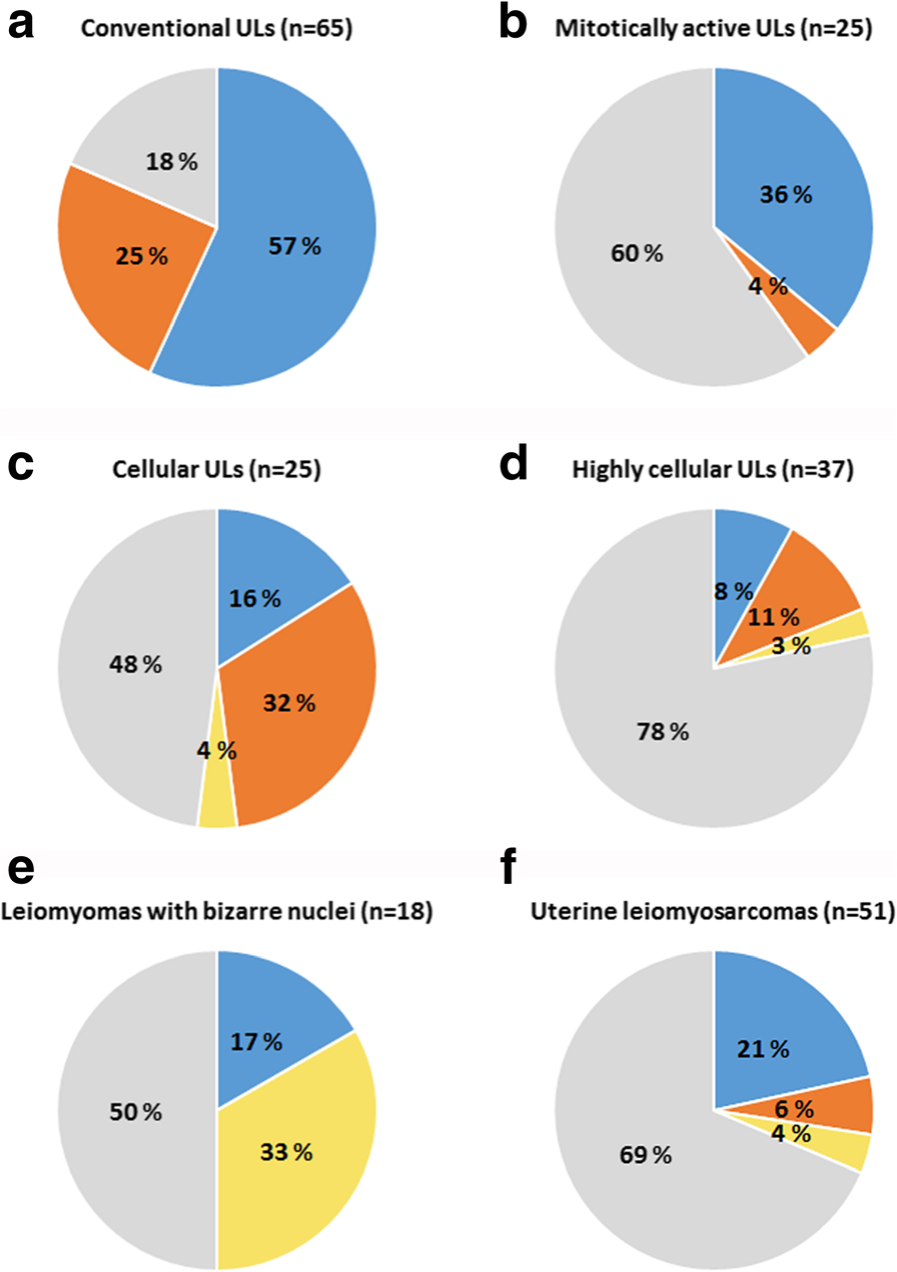
Mutation spectra of uterine smooth muscle tumors. The frequencies of *MED12* mutations, *HMGA2* aberrations, and biallelic *FH* inactivation in conventional ULs **a**, histopathological UL variants **b-e**, and ULMSs **f**. Eleven mitotically active leiomyomas with increased cellularity were included in both mitotically active and cellular/highly cellular leiomyoma subtypes

*MED12* and *HMGA2* alterations accounted for the vast majority of conventional ULs (53/65, 81.5%) (Table 1, Fig. 1, see Additional file 5: Table S2), which is in line with previous literature [11]. The third driver alteration, biallelic *FH* inactivation, is known to be very rare in sporadic conventional ULs [15, 26, 30, 31]. Accordingly, all conventional ULs included in this study were FH proficient [26]. This set of conventional ULs provides thus an appropriate reference series for the other tumor types in this study.

**Table 1:** *MED12* mutations, *HMGA2* aberrations, and biallelic *FH* inactivation in uterine smooth muscle tumors

Smooth muscle tumor subtype	Total	*MED12* mutation positive				*HMGA2* overexpressing				*FH* deficient			
		N	%	*P*-value	95% CI	N	%	*P*-value	95% CI	N	%	*P*-value	95% CI
Conventional	65	37	56.9			16	24.6			0	0		
Histopathological UL variant	94	17	18.1	5.2 × 10^−7^	2.76–13.14	13	13.8	0.10	0.83–5.01	8	8.5	0.02	0.00–0.81
Mitotically active	25	9	36	0.10	0.83–6.93	1	4	0.03	1.07–341.56	0	0		
Leiomyoma with bizarre nuclei	18	3	16.7	3.0 × 10^−3^	1.61–38.18	0	0	0.02	1.24- ∞	6	33.3	4.9 × 10^−5^	0.00–0.19
Cellular	25	4	16	7.3 × 10^−4^	1.98–30.31	8	32	0.60	0.23–2.23	1	4	0.28	0.00–15.00
Highly cellular	37	3	8.1	6.0 × 10^−7^	3.98–81.73	4	10.8	0.12	0.77–11.96	1	2.7	0.36	0.00–22.20
Leiomyosarcoma	51	11	21.6	1.4 × 10^−4^	1.96–12.16	3	5.9	0.01	1.35–29.40	2	3.9	0.19	0.00–4.16

Eleven mitotically active leiomyomas with increased cellularity were included in both mitotically active and cellular/highly cellular leiomyoma subtypes for statistical testing

### Histopathological uterine leiomyoma subtypes

As previously reported, the histopathological UL variants (18.1%, 17/94) harbored significantly fewer *MED12* mutations than the conventional leiomyomas (56.9%, 37/65; *P* = 5.2 × 10^−7^) [12] (Table 1, see Additional file 5: Table S2 and Additional file 6: Table S3). No significant difference was seen in the number of *HMGA2* overexpressing tumors between histopathological UL variants (13.8%, 13/94) and conventional leiomyomas (Table 1, see Additional file 5: Table S2 and Additional file 6: Table S3). Unlike conventional leiomyomas, the histopathological UL variants (8.5%, 8/94) displayed 2SC positivity as a sign of FH deficiency (Table 1, Fig. 1, see Additional file 5: Table S2 and Additional file 6: Table S3).

#### Mitotically active leiomyomas

Mitotically active ULs occur primarily in premenopausal women. Tumors display an increased number of mitotic figures per 10 high power fields, but otherwise present macroscopic and histologic appearances of conventional leiomyomas, lacking cytological atypia and tumor cell necrosis [32, 33]. Hormonal and other external factors in the tumor microenvironment have been associated with this tumor type and are thought to contribute to the elevated number of mitoses. For example, mitotically active ULs are usually diagnosed during the secretory phase of the menstrual cycle [34] and patients using progestin display significantly higher mitotic activity in their leiomyomas compared to control patients [35]. The known leiomyoma driver alterations were observed in 40% of the tumors: *MED12* mutation frequency was slightly, but not significantly, lower in mitotically active ULs (36%, 9/25) compared to conventional leiomyomas (Table 1, Fig. 1, see Additional file 5: Table S2 and Additional file 6: Table S3). Other alterations were very rare as only one tumor showed *HMGA2* overexpression (4%) and none displayed biallelic *FH* inactivation (Table 1, Fig. 1, see Additional file 6: Table S3). Our results suggest that the molecular background of mitotically active ULs slightly differs from that of conventional ULs.

#### Cellular leiomyomas

Cellular leiomyomas present clearly increased cellularity when compared to the surrounding myometrium, lack nuclear atypia and tumor necrosis, and have infrequent mitotic figures [36]. The tumors commonly have an irregular border that merges with the surrounding myometrium. Cytogenetically, these tumors have been shown to display loss of the short arm of chromosome 1, and their transcriptional profile has been suggested to be more similar to leiomyosarcomas than conventional leiomyomas or normal myometrium [37]. Additionally, two small clinical series have reported cellular leiomyomas to associate with aggressive clinical behavior [38, 39]. Based on our results, the most common driver alteration in cellular ULs is *HMGA2* overexpression, which was seen in one third of the tumors (32%, 8/25) (Table 1, Fig. 1, see Additional file 6: Table S3). This was the highest frequency of *HMGA2* overexpression in any tumor type studied. The difference was statistically significant when compared to mitotically active ULs, leiomyomas with bizarre nuclei, or ULMSs. The *MED12* mutation frequency in these tumors was rather low: 16% (4/25) (Table 1, Fig. 1, see Additional file 6: Table S3). Also one cellular UL that presented 2SC positivity (4%) and harbored a potential *FH* mutation in exon 3 (c.321_323del3, p.N107_Q108delinsL) was identified. This three base pair deletion results in the substitution of amino acids N107 and Q108 with leucine (L). Missense mutation affecting the same amino acid (c.320A > C, p.N107T) has been reported in six UK HLRCC families [14], supporting the pathogenicity of the mutation. Overall, the leiomyoma driver alterations were observed in more than half of the cellular ULs (52%, 13/25), but their distribution differed from that observed in conventional tumors.

#### Highly cellular leiomyomas

Highly cellular leiomyomas are characterized with even higher density of cells reminiscent of endometrial stromal tumors, and may be occasionally misdiagnosed as endometrial stromal nodules when well circumscribed [36, 40]. Here, these tumors harbored the lowest number of *MED12* mutations of all tumor types analyzed (8.1%, 3/37) (Table 1, Fig. 1, see Additional file 6: Table S3). Four tumors overexpressed *HMGA2* (10.8%) and one showed 2SC positivity (2.7%). Overall, this tumor subtype displayed the least amount of leiomyoma driver alterations (21.6%, 8/37) suggesting that there are other, still unknown factors underlying the genesis of these lesions.

#### Leiomyomas with bizarre nuclei

Leiomyomas with bizarre nuclei (previously termed ‘atypical leiomyomas’) are characterized by the presence of large cells with eosinophilic cytoplasm and bizarrely shaped, multilobated or -nucleated nuclei. Low mitotic activity and lack of tumor cell necrosis are also typical for these tumors [41, 42]. Interestingly, as many as one third of ULs with bizarre nuclei (33.3%, 6/18; *P* = 4.9 × 10^−5^) displayed 2SC positivity, indicating biallelic *FH* inactivation (Table 1, Fig. 1, see Additional file 6: Table S3). Subsequently, our sequencing efforts identified an *FH* mutation in two of the tumors. One mutation, c.587A > G, p.H196R, has been previously reported in three Finnish HLRCC families [30, 43] and in three isolated cases. The germline origin of the mutation was confirmed also for the patient in this study. Altogether six patients in these previously identified families have been diagnosed with renal cell cancer. Another tumor with bizarre nuclei had a mutation, c.739-2A > C in exon-intron junction preceding exon 6, which likely affects splicing. FH deficiency has been shown to be overrepresented in leiomyomas with bizarre nuclei and some of the morphologic features typical for these tumors have been associated with *FH* mutations [42, 44–47]. Even though morphologic features alone lack robustness to separate sporadic and HLRCC-associated ULs [48–50], the possibility of an underlying germline *FH* mutation should be considered in patients with ULs with bizarre nuclei. Clinical features such as the severity of symptoms, number of tumors within the uterus, age at diagnosis, and possible family history of ULs can be evaluated, and patients should be examined for possible cutaneous leiomyomas. Should additional features suggestive of HLRCC be identified, individuals could be directed to genetic counselling and molecular screening. Subsequently, mutation-positive individuals could be informed on the possible negative effects of HLRCC on fertility, and offered appropriate screening program for renal cell cancer. On the other hand, FH deficiency can also be a somatic event in ULs and may not exclusively indicate HLRCC syndrome, although somatic biallelic *FH* inactivation has only rarely been reported [31, 50]. The most prevalent alterations in conventional ULs were only rarely observed in leiomyomas with bizarre nuclei: *MED12* mutations were identified in three out of 18 tumors (16.7%) and none displayed *HMGA2* overexpression (Table 1, Fig. 1, see Additional file 6: Table S3). Recently, it has been suggested that leiomyomas with bizarre nuclei can be divided into two subtypes based primarily on nuclear features; some tumors show significantly higher rates of HMGA2 immunoreactivity and frequent *MED12* mutations and the others may occasionally be related to *FH* mutations [51]. The high frequency of *FH* inactivation and the low frequency of *MED12* and *HMGA2* alterations distinctly separates these tumors from conventional ULs, other histopathological UL variants, and ULMSs.

### Uterine leiomyosarcomas

Leiomyosarcomas are diagnosed primarily based on cytological atypia, mitotic activity, and tumor cell necrosis, which distinguishes them from benign uterine smooth muscle tumors [52]. Despite well-defined morphological criteria, diagnostic challenges emerge, at times, in daily pathological practice in differentiating ULMSs from histopathological UL subtypes. Leiomyosarcomas are known to harbor complex numerical and structural chromosomal aberrations, as well as mutations in well-known cancer genes *ATRX*, *TP53*, and *RB1* [23, 24, 53]. As previously reported, *MED12* mutations were one of the most common alterations in ULMSs (21.6%, 11/51), though significantly less frequent than in conventional ULs (*P* = 1.4 × 10^−4^) (Table 1, Fig. 1, see Additional file 5: Table S2 and Additional file 7: Table S4). [24, 26, 54]. Also *HMGA2* overexpression was infrequent compared to conventional ULs (5.9%, 3/51, *P* = 0.01) and two tumors showed biallelic *FH* inactivation (3.9%). Although not as common as in conventional ULs, leiomyoma driver alterations were observed in nearly one third of ULMSs (31.4%, 16/51). This suggests that some ULMSs may originate from a leiomyoma precursor, or that the UL driver alterations provide a growth advantage also for these malignant smooth muscle tumors. The progression from a benign UL precursor to a malignant leiomyosarcoma has been proposed also previously based on microscopically visible co-localization of morphologically benign areas within ULMS [13, 55–57]. Furthermore, in some of these cases, identical *MED12* mutations have been identified in both components [13]. Of note, one leiomyosarcoma FFPE block in our sample series showed simultaneous co-localization of a leiomyoma with bizarre nuclei. In this case, both tumors were wild type for all leiomyoma driver alterations.

### Mutual exclusivity

**Fig. 2:**
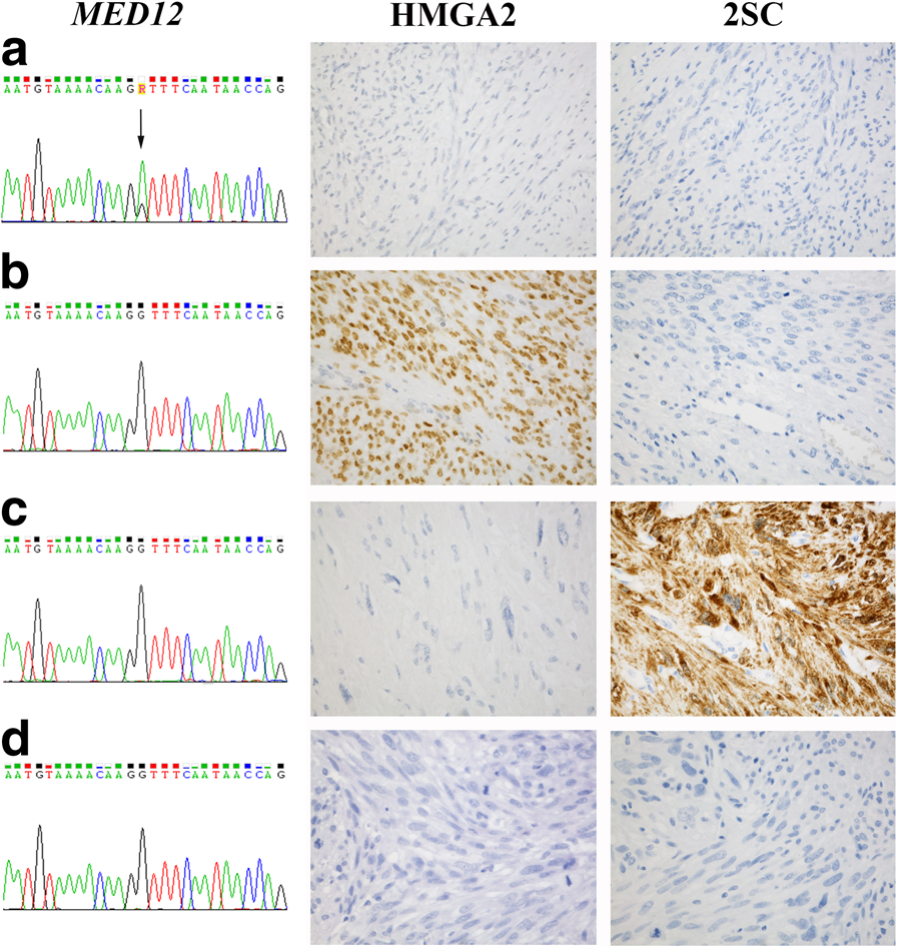
Mutual exclusivity of *MED12* mutations, *HMGA2* overexpression, and biallelic *FH* inactivation. Representative figures of uterine smooth muscle tumors with different molecular genetic features. **a** *MED12* mutation-positive conventional leiomyoma, **b ** cellular leiomyoma with *HMGA2* overexpression, **c** 2SC-positive leiomyoma with bizarre nuclei, and **d** leiomyosarcoma without the known genetic leiomyoma driver aberrations. Antibody stainings are shown with ×40 magnification

Altogether 65 uterine smooth muscle tumors harbored *MED12* mutations, 32 showed *HMGA2* overexpression, and ten 2SC positivity, indicating biallelic *FH* inactivation. None of the tumors displayed simultaneously more than one leiomyoma driver alteration, indicating that *MED12* mutations, *HMGA2* overexpression, and biallelic *FH* inactivation are mutually exclusive (Fig. 2).

### Limitations of the study

The differentiation of uterine smooth muscle tumors may occasionally result in ambiguous cases, which are challenging to diagnose. Although the sample series in this study was large, considering the rarity of histopathological UL subtypes and ULMSs, the classification of tumors based on the leiomyoma driver alterations resulted in rather small subgroups. Also no clinical data were available for the samples. In the future, it would be interesting to study the associations between different tumor subgroups and clinical characteristics in an even larger set of samples. In addition, the fairly modest success rate of *FH* screening may be due to the low quantity and suboptimal quality of FFPE samples, or there might be other kinds of variations that contribute to the genesis of these lesions, such as larger deletions, insertions, or more complex structural alterations, which are not detectable by amplicon sequencing, or alternatively the alterations may be epigenetic or affect non-coding regulatory regions.

## Conclusions

To conclude, 107 out of 210 uterine smooth muscle tumors analyzed harbored one of the three leiomyoma driver alterations. No tumor displayed more than one change, confirming the previous observations [12, 16, 26, 58] that these mutations are mutually exclusive driver alterations. While these alterations were identified in various UL subtypes, their relative frequencies varied considerably. Of particular interest was the high frequency of FH-deficient tumors among leiomyomas with bizarre nuclei; although not an unambiguous indicator for HLRCC, the possibility of this highly penetrant tumor predisposition syndrome should be considered and additional suggestive clinical characteristics evaluated when these tumors are encountered at the clinic. Except for conventional ULs, a significant proportion of all other UL subtypes displayed no driver alterations and additional work is required to reveal the underlying causes in these mutation-negative tumors. Finally, nearly one third of ULMSs displayed one of the three UL driver alterations, emphasizing the role of these aberrations also in the ULMS tumorigenesis. It is clinically relevant to understand the molecular mechanisms driving the tumorigenesis of uterine smooth muscle tumors, as it may lead to improved diagnosis and personalized medical treatments in the future.

## Additional files

Additional file 1: Table S1. Histopathological features of 210 uterine smooth muscle tumors (XLSX 19 kb).

Additional file 2: **Figure S1.** Large deletions in *MED12* exon 2. Three uterine smooth muscle tumors (two conventional leiomyomas and one leiomyosarcoma) harbor large *MED12* exon 2 deletions (PDF 175 kb).

Additional file 3: Figure S2. Scoring of HMGA2 and 2SC antibody stainings. Representative figures of uterine smooth muscle tumors showing different intensities of the immunoreaction for HMGA2 and 2SC (−/(+)/+/++). Antibody stainings are shown with ×40 magnification (PDF 216 kb).

Additional file 4: Figure S3. Representative hematoxylin-eosin stainings of uterine smooth muscle tumor subtypes. HE-stainings are shown with ×40 magnification (PDF 364 kb).

Additional file 5: Table S2. *MED12* exon 1/2 mutation status and results of HMGA2 and 2SC immunohistochemistry of 65 conventional uterine leiomyomas (XLSX 11 kb).

Additional file 6: Table S3 *MED12* exon 1/2 mutation status and results of HMGA2 and 2SC immunohistochemistry of 94 histopathological uterine leiomyoma variants (XLSX 13 kb).

Additional file 7: Table S4. *MED12* exon 1/2 mutation status and results of HMGA2 and 2SC immunohistochemistry of 51 uterine leiomyosarcomas (XLSX 12 kb).

## Abbreviations

2SC: 2-succinocysteine
ATRX: Alpha thalassemia/mental retardation syndrome X-linked
FFPE: Formalin-fixed paraffin-embedded
FH: Fumarate hydratase
HLRCC: Hereditary leiomyomatosis and renal cell cancer
HMGA2: High mobility group AT-hook 2
MED12: Mediator complex subunit 12
RB1: Retinoblastoma 1
TMA: Tissue microarray
TP53: Tumor protein P53
UL: Uterine leiomyoma
ULMS: Uterine leiomyosarcoma
