# Supplementary material for: Characterization of MED12, HMGA2, and FH alterations reveals molecular variability in uterine smooth muscle tumors
Source: Mol Cancer. 2017 Jun 7;16:101. doi: 10.1186/s12943-017-0672-1 (PMC5463371; doi:10.1186/s12943-017-0672-1)
Supplement: Supplementary file 2 — Figure S1. Large deletions in MED12 exon 2. Three uterine smooth muscle tumors (two conventional leiomyomas and one leiomyosarcoma) harbor large MED12 exon 2 deletions (PDF 175 kb). [file 12943_2017_672_MOESM2_ESM.pdf]

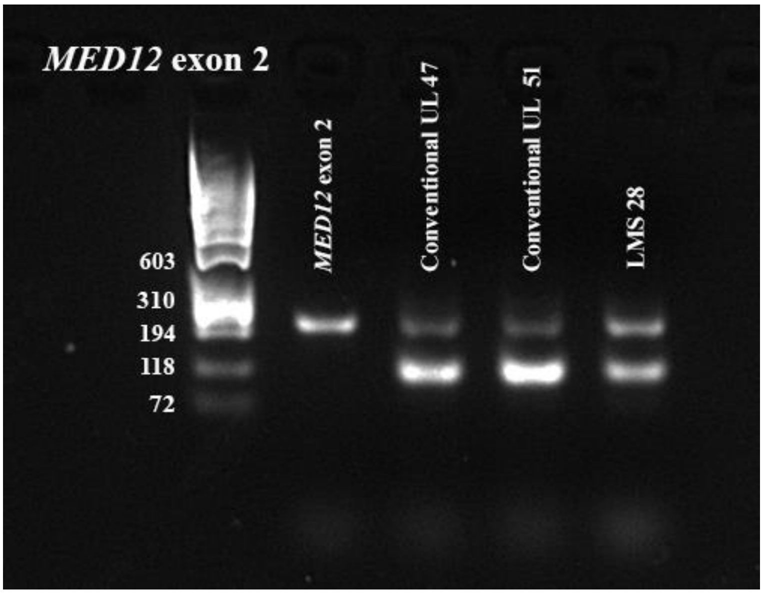

Figure S1. Large deletions in *MED12* exon 2. Three uterine smooth muscle tumors (two conventional leiomyomas and one leiomyosarcoma) harbor large *MED12* exon 2 deletions.
