# Supplementary material for: Characterization of MED12, HMGA2, and FH alterations reveals molecular variability in uterine smooth muscle tumors
Source: Mol Cancer. 2017 Jun 7;16:101. doi: 10.1186/s12943-017-0672-1 (PMC5463371; doi:10.1186/s12943-017-0672-1)
Supplement: Supplementary file 3 — Scoring of HMGA2 and 2SC antibody stainings. Representative figures of uterine smooth muscle tumors showing different intensities of the immunoreaction for HMGA2 and 2SC (−/(+)/+/++). Antibody stainings are shown with ×40 magnification (PDF 216 kb). [file 12943_2017_672_MOESM3_ESM.pdf]

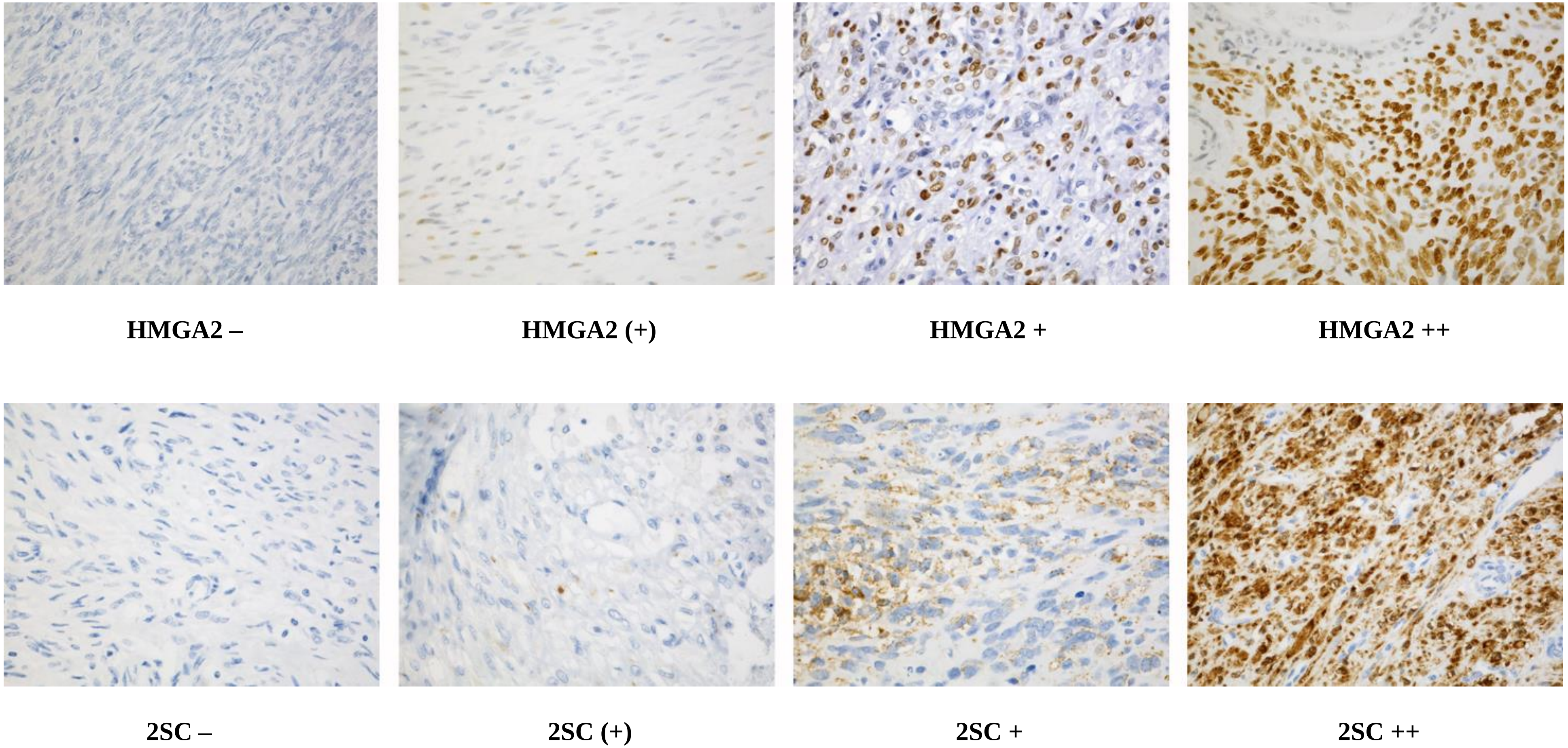

Figure S2. Scoring of HMGA2 and 2SC antibody stainings. Representative figures of uterine smooth muscle tumors showing different intensities of the immunoreaction for HMGA2 and 2SC (-/(+)/+/++). Antibody stainings are shown with ×40 magnification.
