# Supplementary material for: Characterization of MED12, HMGA2, and FH alterations reveals molecular variability in uterine smooth muscle tumors
Source: Mol Cancer. 2017 Jun 7;16:101. doi: 10.1186/s12943-017-0672-1 (PMC5463371; doi:10.1186/s12943-017-0672-1)
Supplement: Supplementary file 4 — Representative hematoxylin-eosin stainings of uterine smooth muscle tumor subtypes. HE-stainings are shown with ×40 magnification (PDF 364 kb). [file 12943_2017_672_MOESM4_ESM.pdf]

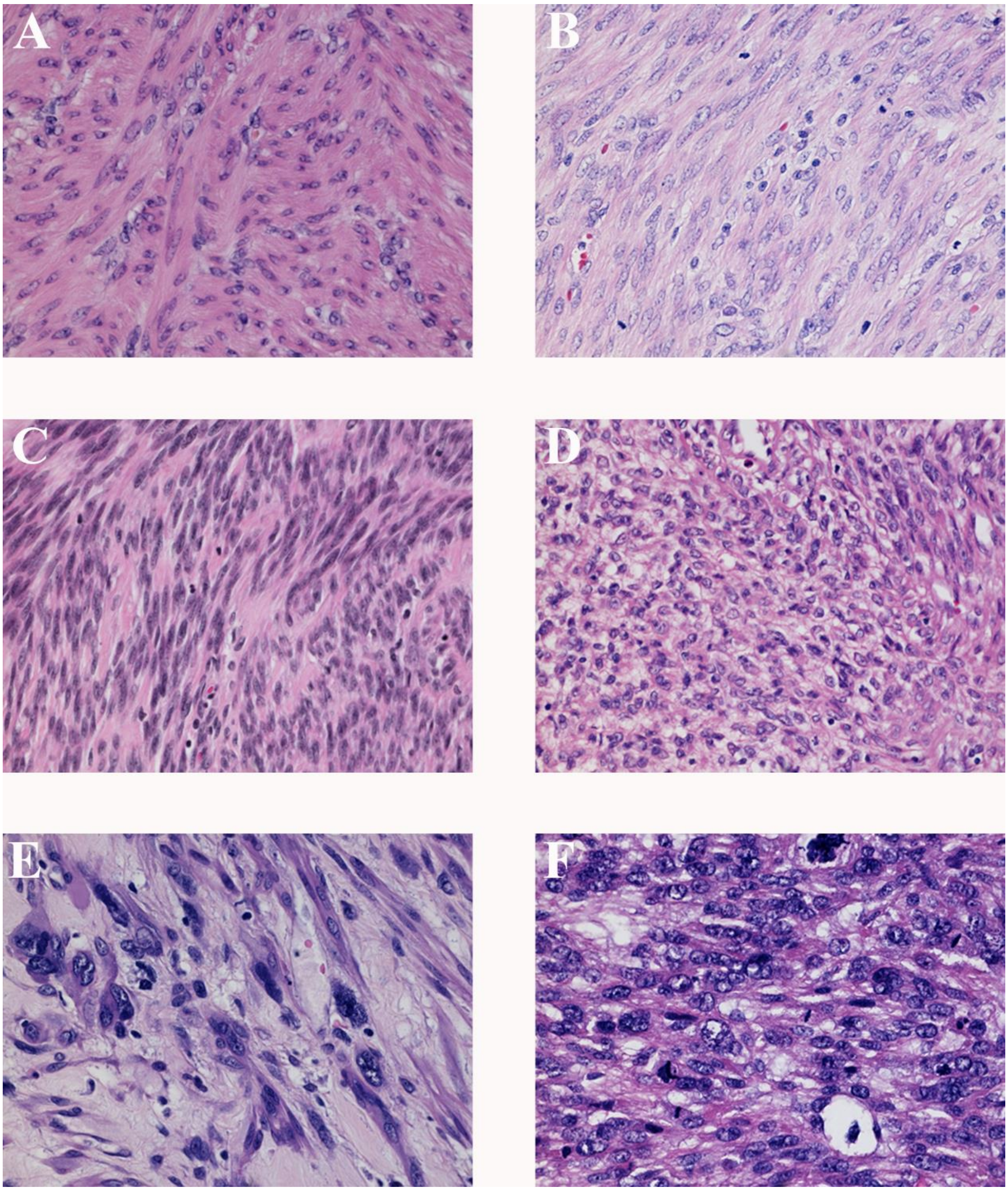

Figure S3. Uterine smooth muscle tumor subtypes. Representative figures of A) conventional, B) mitotically active, C) cellular, D) highly cellular leiomyomas, E) leiomyomas with bizarre nuclei, and F) leiomyosarcomas. HE-stainings are shown with ×40 magnification.
